# Localization of TFIIB binding regions using serial analysis of chromatin occupancy

**DOI:** 10.1186/1471-2199-8-102

**Published:** 2007-11-12

**Authors:** Gregory S Yochum, Veena Rajaraman, Ryan Cleland, Shannon McWeeney

**Affiliations:** 1 Vollum Institute, Oregon Health and Science University, Portland, OR 97239, USA.; 2 Cancer Institute, Oregon Health and Science University, Portland, OR 97239, USA.; 3 Department of Public Health and Preventative Medicine, Division of Biostatistics, Oregon Health and Science University, Portland, OR 97239, USA.

## Abstract

**Background:**

RNA Polymerase II (RNAP II) is recruited to core promoters by the pre-initiation complex (PIC) of general transcription factors. Within the PIC, transcription factor for RNA polymerase IIB (TFIIB) determines the start site of transcription. TFIIB binding has not been localized, genome-wide, in metazoans. Serial analysis of chromatin occupancy (SACO) is an unbiased methodology used to empirically identify transcription factor binding regions. In this report, we use TFIIB and SACO to localize TFIIB binding regions across the rat genome.

**Results:**

A sample of the TFIIB SACO library was sequenced and 12,968 TFIIB genomic signature tags (GSTs) were assigned to the rat genome. GSTs are 20–22 base pair fragments that are derived from TFIIB bound chromatin. TFIIB localized to both non-protein coding and protein-coding loci. For 21% of the 1783 protein-coding genes in this sample of the SACO library, TFIIB binding mapped near the characterized 5' promoter that is upstream of the transcription start site (TSS). However, internal TFIIB binding positions were identified in 57% of the 1783 protein-coding genes. Internal positions are defined as those within an inclusive region greater than 2.5 kb downstream from the 5' TSS and 2.5 kb upstream from the transcription stop. We demonstrate that both TFIIB and TFIID (an additional component of PICs) bound to internal regions using chromatin immunoprecipitation (ChIP). The 5' cap of transcripts associated with internal TFIIB binding positions were identified using a cap-trapping assay. The 5' TSSs for internal transcripts were confirmed by primer extension. Additionally, an analysis of the functional annotation of mouse 3 (FANTOM3) databases indicates that internally initiated transcripts identified by TFIIB SACO in rat are conserved in mouse.

**Conclusion:**

Our findings that TFIIB binding is not restricted to the 5' upstream region indicates that the propensity for PIC to contribute to transcript diversity is far greater than previously appreciated.

## Background

The core promoter is the major regulatory element responsible for determining transcriptional output. The core promoter spans a region of 40–50 bases and encompasses the transcript start site [1]. The core promoter assembles a pre-initiation complex (PIC) of general transcription factors (GTFs) in a step-wise fashion to recruit RNA polymerase II (RNAP II) [2,3]. Reconstitution assays using purified factors demonstrate that TFIIB is required for transcript initiation by RNAP II [4-7]. The importance of TFIIB in transcript initiation was suggested by a co-crystal structure showing that TFIIB positions the coding DNA strand into the active site of RNAP II, thereby ensuring proper TSS selection [8]. Additionally, TFIIB remains at the promoter and does not track with the elongating RNAP II complex [9,10]. Thus, TFIIB is an ideal factor to localize core promoters.

Recently, the isolation and analysis of the mouse transcriptome by the functional annotation of mouse 3 (FANTOM3) consortium indicates that most protein-coding genes produce multiple transcripts [11]. Importantly, for most genes the 5' end of multiple internal transcripts (as identified by the 5' cap structure) localized far downstream of the 5' TSS for the full-length protein-coding transcript. It has been proposed that regulation of internally initiated and variant transcripts may occur through alternative or multiple promoters [12-14].

In this report, we use serial analysis of chromatin occupancy (SACO) to identify TFIIB binding regions in the rat genome. SACO allows an unbiased and genome-wide interrogation of transcription factor binding regions [15,16]. In this method, a transcription factor (in this case TFIIB) is cross-linked to its binding site using formaldehyde, and the DNA-protein complexes are isolated by chromatin immunoprecipitation (ChIP). The DNA is purified from the transcription factor and is then processed into 20–22 bp tags as in long serial analysis of gene expression [17]. In SACO, these tags are referred to as genomic signature tags (GSTs). The GSTs are concatamerized and sub-cloned into a sequencing vector. The concatamers of TFIIB GSTs comprise the SACO library. The TFIIB GSTs are aligned to the genome and only those with unique assignments are further considered. The resolution of SACO is limited by the largest chromatin fragments included in construction of the library (for this library, approximately 2.5 kilobases). Therefore, a conservative estimate is that a TFIIB GST identifies a putative TFIIB binding site within a 2.5 kilobase fragment of chromatin. In the current study, the sequencing and analysis of a sample of the SACO library indicates that internal TFIIB binding positions are a common feature of protein-coding genes.

## Results

### TFIIB SACO library

**Figure 1:**
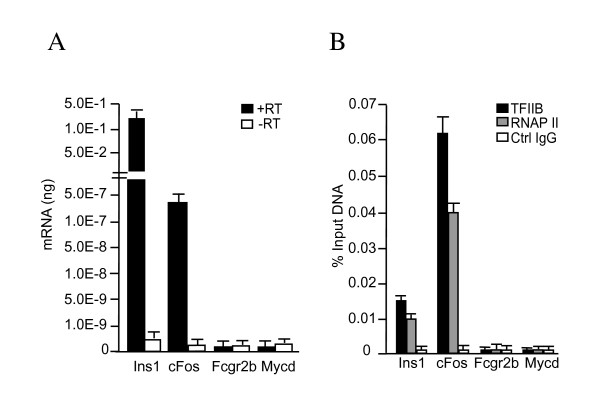
TFIIB binds active genes in Rin-m rat insulinoma cells. (A) RNA was isolated and cDNA was synthesized using reverse transcriptase. Transcript levels of active (*c-Fos *and *Ins1*) and repressed (*Fcgr2b *and *Mycd*) genes were measured by quantitative real-time PCR. Levels of cDNA detected (black bars) were determined using a standard curve generated with purified PCR amplicons. No reverse transcriptase controls (white bars) indicate that signal generated was RNA-dependent. The amount of *Fcgr2b *and *Mycd *was over 500-fold lower than *c-Fos *and therefore represents background amplification in the assay. (B) Real-time PCR quantitation of DNA fragments precipitated in a ChIP assay using 3 μg of TFIIB antibodies (black bars), 3 μg of RNAP II antibodies (gray bars), or 3 μg of Gal4 antibodies as a control IgGs (white bars). The level of immunoprecipitated *Ins1*, *c-Fos*, *Fcgr2b*, and *Mycd *promoters was measured by real-time PCR. The standard curve was derived from PCR reactions using serially diluted input chromatin DNA as the template. Error bars are SEM.

Initially, we tested whether TFIIB binds promoters of active genes in the rat insulinoma cell line, Rin-m. The Rin-m cell line was established from radiation-induced rat islet cell tumor maintained in athymic *nude *mice [18]. Using quantitative reverse transcriptase PCR (qRT-PCR), we found that transcript levels of two genes known to be expressed in Rin-m cells, *insulin *and *cFos *[18,19], were at least two-to-three orders of magnitude higher than repressed genes (the immune cell specific *Fcgr2b *and muscle specific, *myocardin*, *Mycd*) (Figure 1A). TFIIB binding was then tested in ChIP assays using promoter-specific primers. In addition, an antibody directed against the DNA binding domain of the yeast activator Gal4 was used in parallel ChIPs as a control for specificity. TFIIB bound to the *insulin *and *cFos *promoters, but not to the repressed *Fcgr2b *and *Mycd *promoters (Figure 1B). ChIP assays using antibodies specific for RNAP II confirmed that TFIIB associated with transcribed genes. We then constructed a SACO library with DNA isolated from TFIIB ChIPs.

The TFIIB SACO library contains approximately 10^6 ^GSTs indicative of putative TFIIB binding regions. A portion of the library corresponding to 19,204 GSTs was sequenced. Of these, 12,968 (68%) could be assigned to a unique position in the rat genome, similar to the fraction of unique GSTs in previously characterized SACO libraries [15,16]. We mapped the distribution of TFIIB GSTs on each rat chromosome to determine whether this set of identified regions represents an unbiased sample (see Additional file 1). The chromosomes contain between 210 and 1416 TFIIB GSTs. The average number of TFIIB binding sites per megabase of DNA ranges from approximately two on the X chromosome to eight on chromosome 16. We then focused on a sub-population of the library that contains 2481 distinct TFIIB GSTs localizing to 1783 protein-coding genes in the reference sequence (RefSeq) database. An alignment of TFIIB GSTs and corresponding RefSeq genes on chromosome 10 demonstrate that the entire length of the chromosome is represented in the SACO library (see Additional file 2). A similar representation was present at other chromosomes examined (data not shown). Therefore, chromosome size and position do not appear to bias TFIIB localizations identified in our library.

### TFIIB binding occurs internally as well as 5' and 3'

**Figure 2:**
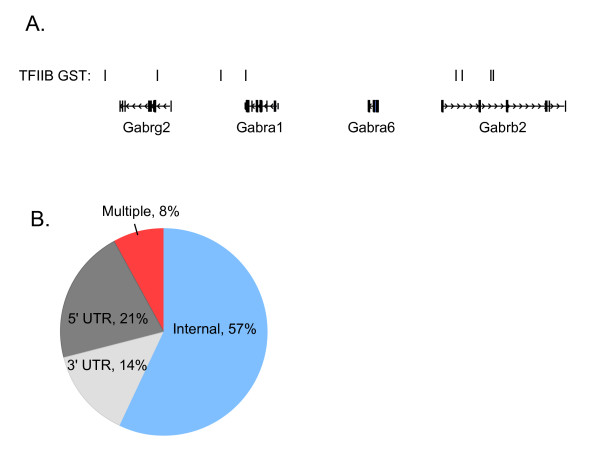
Distribution of TFIIB GSTs relative to RefSeq gene annotations. (A) A segment of chromosome 10 depicting internal and 3' localization relative to four RefSeq genes from the *Gamma-aminobutyric acid A receptor *(*Gabrs*) subfamily. Rectangles are exons and horizontal lines introns with hatch marks indicating the direction of transcription. (B) Locations of TFIIB GSTs relative to each of the 1783 RefSeq genes identified in the SACO library. 5' UTR; 5' untranslated region, 3' UTR; 3' untranslated region, Internal; Internal region, Multiple; the RefSeq gene contains more than one TFIIB GST. RefSeq genes comprising the "multiple" category contain TFIIB GSTs that localize to 5' and internal regions, 3' and internal regions, 5' and 3' regions, or 5', 3' and internal regions.

A key question of interest was to identify the position of TFIIB GSTs relative to RefSeq gene boundaries. We found that TFIIB occupied internal and 3' positions in addition to the more traditional 5' promoter region. One example of the distribution of putative TFIIB binding sites is shown in Figure 2A for genes of the *gamma-aminobutyric acid A receptor *subfamily (*Gabrs*). We then localized all TFIIB GSTs in the library relative to RefSeq gene boundaries. TFIIB GSTs were classified as 5' promoter associated if they were found in an inclusive region 2.5 kb outside and 2.5 kb inside the transcript start site (TSS), 3' promoter associated if they were within a region 2.5 kb outside and 2.5 kb inside the 3' end, and internal if they were greater than 2.5 kb inside either the 5' or 3' ends of gene boundaries. The 2.5 kb limit was chosen based upon agarose gel determination of the uppermost size of the chromatin fragments included in library construction (see Additional file 3). There is a total of 1783 RefSeq genes with uniquely assigned GSTs in the TFIIB SACO library. 21% of the genes localized TFIIB binding to the 5' UTR relative to the characterized TSS of the RefSeq gene (Figure 2B). 14% had 3' UTR localizations thereby identifying potential promoters that drive antisense transcripts. Surprisingly, most genes (57%) contained internal TFIIB binding positions. Moreover, 8% of the RefSeq genes contained multiple TFIIB binding regions; most with an internal position. Therefore, internal TFIIB binding is a common characteristic of protein-coding genes.

### Confirmation of internal PIC binding and internal transcripts

**Figure 3:**
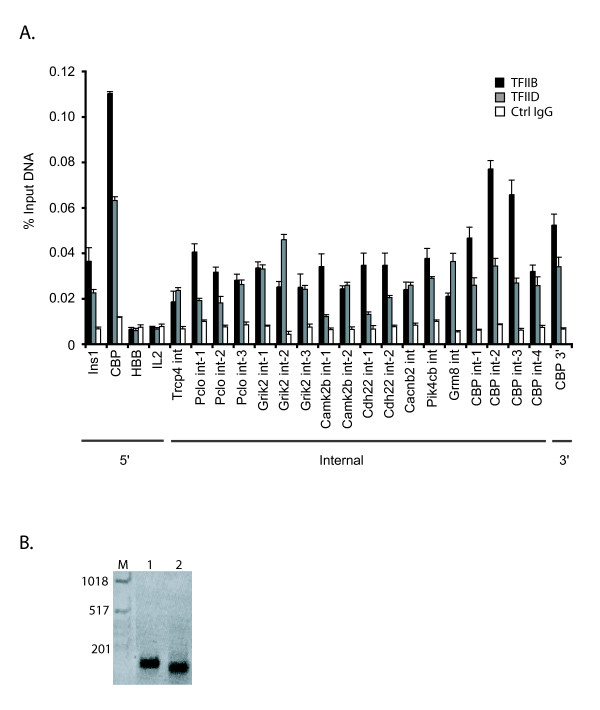
TFIIB and TFIID bind internal positions in RefSeq genes and are associated with internally initiated transcripts. (A) Real-time PCR quantitation of DNA fragments precipitated in a ChIP assay using 3 μg TFIIB antibody (black bars), 10 μl of TFIID antibody (gray bars) or 3 μg Gal4 antibody as a control IgG (white bars). Regions interrogated correspond to a subset of genes with internal sites that were chosen at random. The levels of *Ins1 *and *CBP *5' promoters precipitated are included as controls. Negative regions included the repressed *HBB *and *IL2 *genes. Data is presented as percent input and error is SEM. 5', internal, and 3' refer to the positions of the TFIIB GST relative to RefSeq gene boundaries. (B) An agarose gel of the amplified transcript from an internally initiated *CBP *transcript identified by a modified cap trapping procedure (see methods). Two independent PCR clones (lanes 1 and 2) mapped the start site to nucleotide 11687697 on chromosome 10.

The high percentage of internal TFIIB GSTs prompted us to further validate whether these TFIIB sites identified promoter regions; *i.e*. whether they were associated with transcriptional start sites. First, we performed ChIP assays to confirm TFIIB binding to 18 internal positions of 9 genes demarcated by TFIIB GSTs. TFIIB bound each of the 18 internal positions examined (Figure 3A). TFIIB also bound a region at the 3' end of *CBP*. To test whether the PIC was recruited to the internal TFIIB positions, we assayed for TFIID binding by ChIP. TFIID plays a role in promoter recognition within the PIC [3]. Like TFIIB, TFIID bound each internal position tested (Figure 3A). The levels of TFIIB and TFIID binding to the internal and 3' positions were similar to that at the 5' promoter of *Ins1 *and *CBP*. No significant binding for TFIIB or TFIID was detected at the repressed *HBB *and *IL2 *genes.

Next, we mapped the 5' ends of 18 internal transcripts for the 9 genes in Figure 3A with internal TFIIB binding regions. Using a modified cap trap assay [20,21], we found that the 5' ends of all 18 internal transcripts surveyed were associated with TFIIB GSTs. A representative agarose gel is shown for the cap-trapped TSS of an internal transcript from *CBP *(Figure 3B). This indicates that the internal TFIIB positions are associated with TSSs. We also mapped the TSS for an antisense *CBP *transcript identified by proximity to a 3' TFIIB GST.

**Figure 4:**
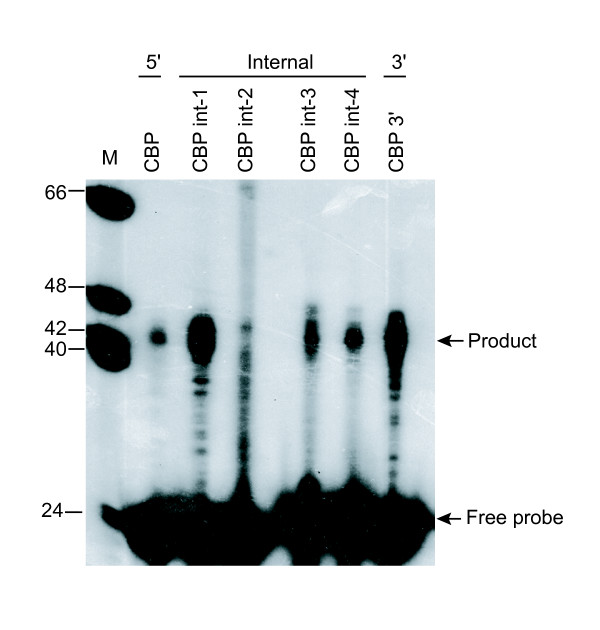
Primer extension analysis of transcripts derived from the rat CBP locus. RNA was isolated from Rin-m cells and primer extension analysis was performed based on TSSs determined by cap-trapping. Products were resolved by denaturing polyacrylamide gel electrophoresis. Marker (M): End labeled Φ X174 Hinf 1 digested DNA. 5', internal, and 3' refer to the position of the TFIIB GST and TSS relative to CBP gene boundaries.

The cap-trapping experiments confirmed that transcripts were initiated at internal positions within the protein-coding genes identified by TFIIB SACO. We then used primer extension to confirm the cap-trapping results with an alternative method. Primer extension requires an oligonucleotide that anneals downstream of a TSS to prime a cDNA synthesis reaction. The reverse transcriptase extends the cDNA until it reaches the 5' end of the transcript. The resulting products are ultimately run on a polyacrylamide gel. We isolated five capped transcripts associated with TFIIB GSTs for *CBP*. Four of the GSTs were associated with internal-sense transcripts and one with an antisense transcript. For the primer extension assay, the characterized 5' promoter was included as a control. We detected transcripts that initiated from the internal and antisense promoters, in addition to the known 5' promoter (Figure 4). Moreover, *CBP *int-1 and the *CBP *3' antisense transcript appeared to be more abundant that the *CBP *transcript derived from the 5' promoter.

### Internal promoters are evolutionarily conserved

**Figure 5:**
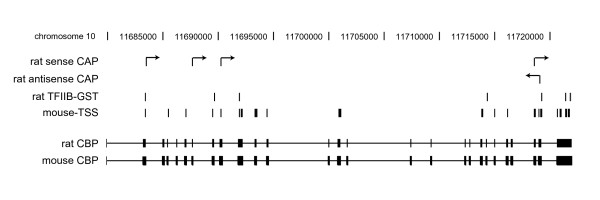
Internal transcripts associated with TFIIB GSTs in rat are conserved in mouse. Diagram depicting the spatial relationship between TFIIB GSTs and the 5' end of sense and antisense transcripts determined by cap-trapping. The 3' portion of rat *CBP *is shown in black with exons as rectangles and introns as a horizontal line. Black vertical lines indicate the position of the feature listed. Black vertical lines with an arrow conferring direction of the transcript indicate the 5' nucleotides of cap-trapped transcripts. Rat sense and antisense CAP are the positions of the experimentally capped transcripts. Mouse-TSS are the positions of the 5' nucleotides of mouse *CBP *transcripts identified by cap analysis of gene expression (CAGE). The mouse TSS data was obtained from the FANTOM3 consortium [11].

We then utilized data from the FANTOM3 consortium as an independent source to validate our findings and to determine whether the internal transcripts we identified in rat are conserved in mouse. The FANTOM3 consortium recently determined the 5' and 3' boundaries for over 180,000 unique RNAs comprising the mouse transcriptome [11]. Using NCBI's Homologene, we identified 1462 RefSeq mouse homologs of the rat genes in our TFIIB SACO library. Of the mouse homologs, 1284 (88%) had cap analysis of gene expression (CAGE) evidence for internal TSSs. We then positionally co-aligned the rat TFIIB GSTs identified in our TFIIB SACO screen with the mouse TSSs reported by the FANTOM3 consortium. Overall, with respect to distance, 84% of the GSTs were within 2.5 kb of a TSS (80% of these sites were within 750 bp). To illustrate this co-alignment, we considered *CBP *as an example (Figure 5). Rat *CBP *had the most TFIIB GSTs and the highest number of experimentally confirmed TSS by our modified cap-trapping method. The rat TFIIB GSTs and the rat TSS that we identified in *CBP *were mapped to mouse *CBP*. We then overlaid transcript start positions within mouse *CBP *identified by FANTOM3. The positions of internal TSS in mouse *CBP *co-align with TFIIB GSTs and TSS in rat *CBP*, suggesting that the internal promoters are conserved across the two species. Together, this analysis suggests that full-length protein-coding transcripts may comprise only a fraction of the transcripts derived from a gene.

## Discussion

The TFIIB SACO approach described in this report allowed us to ascertain experimentally the prevalence of TFIIB binding and PIC localization among protein-coding genes. We found that the majority of protein-coding genes were characterized by internal TFIIB binding (Figure 2). Several lines of evidence demonstrate that the internal positions represent core promoters. First, repeat ChIP assays performed using antibodies against TFIIB and TFIID demonstrate that each of the 18 internal sites of 9 RefSeq genes chosen at random was occupied by PIC *in vivo *(Figure 3). Second, we experimentally isolated eighteen 5' capped transcripts closely associated with internal TFIIB positions from nine RefSeq genes. Third, 88% of the mouse homologs identified in our screen had CAGE evidence for internal TSSs [11]. These findings in conjunction with the earlier FANTOM3 transcript analysis reiterate that alternative promoters are a common feature of protein-coding genes. Thus, our localization of PIC via TFIIB binding suggests that positional diversity of core promoters has been underestimated.

In the last few years, we have begun to understand the complexity and diversity of core promoters that reside in the mammalian genome [22]. This is in stark contrast to the early emphasis on three key motif elements that defined a core promoter: a TATA box, an initiator (Inr) element, and a downstream promoter element (DPE). In comparison with *D. melanogaster *(in which much of the core promoter architecture was originally characterized), mammalian core promoters less frequently contain TATA boxes, have a lower frequency of pairing TATA with Inr elements, and many promoters, including those within CpG islands, appear to lack all three of these core elements [23]. Two TFIIB recognition elements (BREs) have been demonstrated to mediate TFIIB binding within core promoters. BRE^u ^is upstream of the TATA box and BRE^d ^is downstream. It is known that both the BREu and the BREd modulate promoter activity but that this effect is dependent on the specific composition of elements present within a core promoter [24]. This core promoter heterogeneity has made motif analysis of promoters challenging. A recent study by Kim et. al. demonstrates this issue [25]. Using a TAF1 antibody to identify core promoters using a ChIP-coupled microarray, it was discovered that the TATA box was not significantly enriched among 10,567 active promoters in fibroblast cells. Whether or not there will be a uniform code of motifs that determine whether a stretch of DNA is predicted to function as a core promoter await further experimentation. Informative motif analysis of genome-wide studies, like Kim et. al. [25] or this study, will likely require experimental assays to categorize the vast array of sequences into putative classes of core promoters.

The most significant finding of our study is that TFIIB binding localizes to positions other than the 5' promoter of protein-coding genes. While the strong co-occurrence of TFIIB GSTs with transcript start sites identified by the FANTOM3 group suggest that these alternative positions identify promoters, TFIIB has been shown to have additional roles outside of transcript start site selection. Singh and Hampsey recently reported that TFIIB binding to terminator sequences located downstream of protein coding regions [26]. Instead of functioning as a core promoter to drive an anti-sense transcript, TFIIB bound at this position communicated with the 5' promoter via a loop structure. The authors proposed that this loop between the 5' promoter and 3' terminator plays a role in transcript re-initiation. It will be of interest to determine whether TFIIB binding at the 3' end of protein-coding genes identified in our SACO screen, similarly cooperates with the 5' promoter.

## Conclusion

The results presented here provide the first genome-wide mapping of TFIIB binding in a metazoan. Because TFIIB is required for RNAP II dependent transcription, our SACO screen provides an unbiased localization of promoter elements. Our finding that TFIIB occupation of internal regions is common within genes suggests that the full-length protein-coding transcripts may in fact represent a fraction of the genetic output from these loci. Identification of evolutionarily conserved internal promoters suggests that adjacent transcripts may be subjected to regulation that is independent of the 5' untranslated region and promoter elements. Clearly we are only at the very beginnings with our understanding of the transcriptome and its regulation in higher eukaryotes.

## Methods

### Cell culture

Rin-m cells (ATCC #CRL-2057), passage 5–10, were grown in RPMI 1640 (Invitrogen) supplemented with 10% FBS (Hyclone), 100 units/ml penicillin, 100 units/ml streptomycin, and 5 mM L-glutamine. Cells were maintained at 37°C and 5% CO_2 _and were 70–75% confluent at the time of harvesting.

### Chromatin immunoprecipitation

Antibodies used for ChIP included: 3 μg TFIIB c18 (Santa Cruz, sc-225), 3 μg RNAP II (Santa Cruz, sc-9001), 10 μl TFIID (Upstate Cell Signaling solutions, 06-241), and 3 μg Gal4 (Santa Cruz, sc-577). According to the manufacturer the TFIID antibody is directed against TBP. ChIP assays contained 5 × 10^6 ^cells and were conducted as reported [21,27]. Briefly, chromatin in formaldehyde-fixed lysates was sonicated to an average size of approximately 750 bp using a sonic dismembrator 60 (Fisher Scientific). Sonication was conducted for 5 × 20 sec, output 7, with 1 min intermittent rest periods. Lysates were clarified by centrifugation at 20,000 × g for 10 min at 4°C and then incubated with primary antibody overnight at 4°C. Immunocomplexes captured with bovine serum albumin/glycogen-blocked protein A sepharose (Repligen) were washed, and precipitated DNA fragments were isolated with 10% w/v Chelex-100 (BioRad). Isolated fragments were quantified by real-time PCR as previously described [15]. Primers were designed using Primer3 software from the Massachusetts Institute of Technology (MIT). Primers were synthesized at Integrated DNA Technologies (IDT), and sequences are available upon request.

### SACO library

For a complete protocol for constructing a SACO library see [15,21]. The TFIIB SACO library was subcloned into the Sph1 site of pZERO2 (Invitrogen). A second Sph1 site in the kanamycin resistance gene in pZERO-2 was mutated using the Quikchange mutagenesis kit (Stratagene) prior to subcloning of the concatamers. The complete list of genomic targets identified is available upon request.

### Sequencing

Sequencing was performed at High-Throughput Sequencing Solutions (Seattle, WA).

### Quantitative RT-PCR

5 × 10^6 ^Rin-m cells were lysed using a Qiashredder column (Qiagen) and RNA was isolated using the RNeasy kit (Qiagen). Genomic DNA was removed using the DNA free kit (Ambion). First strand cDNA was synthesized from 1 μg of DNA free RNA using a random hexamer primer (Invitrogen) and Superscript III Reverse Transcriptase (Invitrogen). Reactions lacking reverse transcriptase were analyzed to survey the presence of genomic DNA. Real-time PCR was conducted with 50 ng of cDNA and the assay was capable of detecting 10–50 copies of target cDNA.

### Mapping of 5' capped nucleotides

A modification of the first choice RNA mediated rapid amplification of cDNA ends kit (RLM-RACE, Ambion) was used to identify 5' capped transcripts associated with TFIIB GSTs as reported [20,21]. Total RNA was isolated from Rin-m cells with TRIzol (Invitrogen) and 10 μg was treated with calf intestinal phosphatase in a 20 μl reaction at 37°C for 60 min to remove 5' phosphates of contaminating nucleic acids. The 5' cap structure of the remaining RNA was removed by treatment with tobacco acid pyrophosphatase (TAP) in a 20 μl reaction at 37°C for 60 min which leaves a free 5' monophosphate. An oligonucleotide containing two nested primer sites was ligated to the 5' end with T4 RNA ligase in a 20 μl reaction at 42°C for 60 min. First strand cDNA synthesis with M-MLV reverse transcriptase and random decamers was performed in a 20 μl reaction at 42°C for 60 min. The cDNA was was amplified with 10 μM of a primer to the 5' cassette, and 10 μM of a gene-specific primer designed 700–1000 bp away from the TFIIB tag. The products were further amplified by nested PCR using a second internal primer to the 5' cassette and a second gene specific primer. The amplicons were gel purified, cloned into TOPO PCR2.1 (Invitrogen) and analyzed by sequencing.

### Primer extension

Primer extension was performed with the primer extension/AMV reverse transcriptase system (Promega). Primers were designed approximately 50 bp downstream of the 5' capped nucleotide of *CBP *were end-labeled with ^32^P-γ-ATP (Perkin Elmer) using T4 polynucleotide kinase (NEB). Unincorporated ^32^P-γ-ATP was removed using a NucAway spin column (Ambion) and specific activity of the probes was determined using a 2200CA liquid scintillation analyzer (Packard). Total RNA was isolated using TRIzol (Invitrogen) and 10 μg was annealed to the radiolabeled primer at 58°C for one hour. cDNA was extended using AMV reverse transcriptase (Promega). Products were resolved on a 7 M urea/1× TBE (89 mM Tris base, 2 mM EDTA, 89 mM boric acid)/8% polyacrylamide denaturing gel using a sequencing gel apparatus (Gibco BRL, Invitrogen). An end-labeled Φ× *HinfI *digested ladder was included for size reference.

### Computational analysis

Initial processing and placement of the GSTs followed the pipeline established for our previous SACO libraries [15,16]. Chromosome locations, start, end, and orientation of rat RefSeq features aligned to the rn3 build of the rat genome were obtained from the UCSC annotation database. The 1783 RefSeq features that were identified to be associated with putative internal TFIIB binding sites were mapped to their homologs in mouse using Homologene. Genomic coordinates and orientation for mouse RefSeq transcripts were obtained from the FANTOM3 build. The annotated 5' ends of transcripts were obtained from the boundary_set dataset from the FANTOM3 study [11]. The boundary_set dataset was also used to locate internal TSSs for the mouse homologs. All TSS with evidence of 1 or more CAGE tags (or had a reliability of 1 assigned by FANTOM3 curators) were selected for further analysis. Data and annotation from FANTOM3 were based on the mm5 build of the mouse genome.

## Authors' contributions

GSY and SM conceived the experimental design. GSY constructed the SACO library, performed the chromatin immunoprecipitations, cap-trapping, and primer extensions. RC assisted with validation of the SACO library. VR and SM positioned TFIIB GSTs relative to individual chromosomes and to RefSeq gene annotation. VR and SM also performed the analysis with the mouse TSS data deposited by the FANTOM3 consortium. GSY and SM wrote the manuscript. All authors gave final approval of the version to be published.

## Supplementary Material

Additional file 1 **Distribution of TFIIB GSTs across rat chromosomes**. A list of mapped GSTs, corresponding chromosomes, and number of GSTs identified per megabase (Mb) of DNA. Click here for file

Additional file 2 **Location of TFIIB GSTs and associated RefSeq genes on chomosome 10**. Gray bars on the chromosomes are the TFIIB GSTs within 2.5 kb of a RefSeq gene (indicated above the chromosome). Click here for file

Additional file 3 **Sonicated chromatin input for SACO library construction**. Ethidium bromide stained 1% agarose gel of sonicated chromatin used to generate the TFIIB SACO library. L: 1 kb DNA ladder (Invitrogen). The sizes of some ladder fragments are indicated in nucleotide bases. Click here for file
