# Supplementary material for: Localization of TFIIB binding regions using serial analysis of chromatin occupancy
Source: BMC Mol Biol. 2007 Nov 12;8:102. doi: 10.1186/1471-2199-8-102 (PMC2211499; doi:10.1186/1471-2199-8-102)
Supplement: Additional file 1 — Distribution of TFIIB GSTs across rat chromosomes. A list of mapped GSTs, corresponding chromosomes, and number of GSTs identified per megabase (Mb) of DNA. [file 1471-2199-8-102-S1.pdf]

Chromosome (size in Mb)	Mapped TIIB GSTs	GST/ Mb
chr:1 (267.4 Mb)	1416	5.3
chr:2 (255.3 Mb)	1133	4.4
chr:3 (166.0 Mb)	864	5.2
chr:4 (186.1 Mb)	809	4.3
chr:5 (171.6 Mb)	764	4.5
chr:6 (134.2 Mb)	810	6.0
chr:7 (141.6 Mb)	1040	7.3
chr:8 (126.8 Mb)	576	4.5
chr:9 (109.5 Mb)	396	3.6
chr:10 (101.2 Mb)	788	7.8
chr:11 (73.6 Mb)	389	5.3
chr:12 (43.6 Mb)	324	7.4
chr:13 (75.1 Mb)	469	6.2
chr:14 (105.7 Mb)	510	4.8
chr:15 (106.1 Mb)	304	2.9
chr:16 (76.6 Mb)	599	7.8
chr:17 (91.0 Mb)	432	4.8
chr:18 (84.7 Mb)	392	4.6
chr:19 (56.4 Mb)	210	3.7
chr:20 (50.6 Mb)	321	6.4
chr:X (130.8 Mb)	270	2.1
