# Supplementary figures and images for: Localization of TFIIB binding regions using serial analysis of chromatin occupancy
Source: BMC Mol Biol. 2007 Nov 12;8:102. doi: 10.1186/1471-2199-8-102 (PMC2211499; doi:10.1186/1471-2199-8-102)

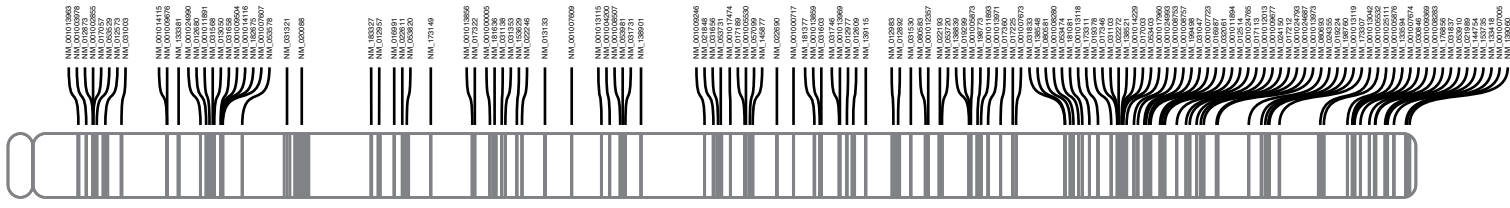

Supplement: Additional file 2 — Location of TFIIB GSTs and associated RefSeq genes on chomosome 10. Gray bars on the chromosomes are the TFIIB GSTs within 2.5 kb of a RefSeq gene (indicated above the chromosome). [file 1471-2199-8-102-S2.pdf]

L

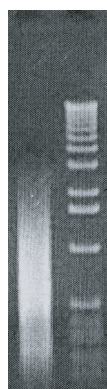

3054

2036

1636

1018

506

298

Supplement: Additional file 3 — Sonicated chromatin input for SACO library construction. Ethidium bromide stained 1% agarose gel of sonicated chromatin used to generate the TFIIB SACO library. L: 1 kb DNA ladder (Invitrogen). The sizes of some ladder fragments are indicated in nucleotide bases. [file 1471-2199-8-102-S3.pdf]
